# Relationship between Community Drug Administration Strategy and Changes in Trachoma Prevalence, 2007 to 2013

**DOI:** 10.1371/journal.pntd.0004810

**Published:** 2016-07-06

**Authors:** Bette Liu, Carleigh Cowling, Andrew Hayen, Gabrielle Watt, Donna B. Mak, Stephen Lambert, Hugh Taylor, John M. Kaldor

**Affiliations:** 1 School of Public Health and Community Medicine, UNSW, Sydney, Australia; 2 The Kirby Institute, UNSW, Sydney, Australia; 3 Centre for Disease Control, Northern Territory Department of Health, Darwin, Australia; 4 Communicable Disease Control Directorate, Health Department of Western Australia, Perth, Australia; 5 School of Medicine, University of Notre Dame Australia, Fremantle, Australia; 6 UQ Child Health Research Centre, The University of Queensland, Brisbane, Australia; 7 Melbourne School of Population and Global Health, Carlton, Melbourne, Australia; University of Queensland School of Veterinary Science, AUSTRALIA

## Abstract

**Background:**

Australia is the only high income country with persisting endemic trachoma. A national control program involving mass drug administration with oral azithromycin, in place since 2006, has some characteristics which differ from programs in low income settings, particularly in regard to the use of a wider range of treatment strategies, and more regular assessments of community prevalence. We aimed to examine the association between treatment strategies and trachoma prevalence.

**Methods:**

Through the national surveillance program, annual data from 2007–2013 were collected on trachoma prevalence and treatment with oral azithromycin in children aged 5–9 years from three Australian regions with endemic trachoma. Communities were classified for each year according to one of four trachoma treatment strategies implemented (no treatment, active cases only, household and community-wide). We estimated the change in trachoma prevalence between sequential pairs of years and across multiple years according to treatment strategy using random-effects meta-analyses.

**Findings:**

Over the study period, 182 unique remote Aboriginal communities had 881 annual records of both trachoma prevalence and treatment. From the analysis of pairs of years, the greatest annual fall in trachoma prevalence was in communities implementing community-wide strategies, with yearly absolute reductions ranging from -8% (95%CI -17% to 1%) to -31% (-26% to -37%); these communities also had the highest baseline trachoma prevalence (15.4%-43.9%). Restricting analyses to communities with moderate trachoma prevalence (5–19%) at initial measurement, and comparing community trachoma prevalence from the first to the last year of available data for the community, both community-wide and more targeted treatment strategies were associated with similar absolute reductions (-11% [-8% to -13%] and -7% [-5% to -10%] respectively). Results were similar stratified by region.

**Interpretation:**

Consistent with previous research, community-wide administration of azithromycin reduces trachoma prevalence. Our observation that less intensive treatment with a ‘household’ strategy in moderate prevalence communities (5-<20%) is associated with similar reductions in prevalence over time, will require confirmation in other settings if it is to be used as a basis for changes in control strategies.

## Author Summary

Australia is the only high income country with persisting endemic trachoma and a national control program has been in place since 2006. The program involves annual screening of children for trachoma in communities designated to be at high risk of disease and treatment of those affected with the antibiotic azithromycin. Depending on the level of trachoma detected in children, antibiotic treatment is also given to households and other community members. We used data collected annually from 2007 to 2013 to examine what effect the extent of azithromycin treatment had on subsequent levels of trachoma in children aged 5–9 years. We found that in communities with high levels of trachoma, when all community members received azithromycin (community-wide treatment), the greatest reduction in trachoma level was achieved. However in communities with moderate levels of trachoma, using either community-wide treatment or more targeted (household) treatment resulted in equivalent reductions in trachoma. This observation needs to be confirmed in other studies before changes to current recommendations regarding trachoma control strategies are considered.

## Introduction

Trachoma, caused by serotypes of *Chlamydia trachomatis* is a major cause of blindness globally.[1] In 1997 The Alliance for the Global Elimination of Blinding Trachoma by 2020 (GET 2020) initiative was launched. Supported by the World Health Organization (WHO), the alliance promotes its goal of elimination through the SAFE strategy, with its key components of surgery to correct trichiasis (S), antibiotic treatment (A), facial cleanliness (F) and environmental improvements (E).[1] Randomised controlled trials have shown that antibiotics, either topical or oral, are effective for treatment.[2] There is a more limited body of trial evidence that has been used to support the strategy of mass drug administration (MDA), or whole community treatment, which is one of the main components of the SAFE strategy in many countries. There have been few comparisons of alternative community treatment strategies, and relatively limited follow up studies of long term trends following implementation of prevention programs.[3,4] Evidence of effective treatment strategies across a range of prevalence settings will become increasingly important as more countries approach the goal of trachoma elimination.

Australia is the only high-income country with endemic trachoma.[5] The disease occurs primarily in remote Aboriginal communities in three Australian jurisdictions, the Northern Territory (NT), South Australia (SA), and Western Australia (WA), although it has also been identified in Queensland and New South Wales.[6,7] In 2013 overall prevalence among children aged 5–9 years in endemic areas was estimated to be 4% with substantial variation between communities; an estimated 50% of communities had no clinically detectable trachoma and 8% had hyperendemic levels (>20%).[8]

Since 2006 the Australian Government has funded control programs based on regular mapping of trachoma prevalence in endemic areas. Trachoma management has been based on guidelines first endorsed in 2006 [9] and revised in 2014.[10] Unlike the WHO guidelines, the 2006 Australian guidelines recommended screening every community considered at risk annually, regardless of trachoma prevalence, as well as a tiered approach to antibiotic treatment depending on trachoma prevalence (see Table 1). Australia therefore has an opportunity to examine the impact of different treatment strategies, in more detail than has been possible in other trachoma endemic settings, where only MDA has been used, and prevalence is generally monitored at much longer intervals. We report here an analysis based on routinely collected trachoma prevalence data over seven years in Australia’s endemic areas. These data have the potential to inform trachoma control programs both in Australia and internationally.

**Table 1:** Comparison of WHO and Australian 2006 guidelines for trachoma management.

	WHO[5]	2006 CDNA Guidelines for the public health management of trachoma in Australia[9]
Target age group	1–9 years of age	Minimum 5–9 years of age. 1–4 and 10–14 screened if resources available. Prevalence is calculated as % in 1–9 years of age.
=> 20% trachoma prevalence	Mass treatment (MDA) for all members of the district aged 6 months and older for 3 years. Repeat survey. Continue MDA until <5% trachoma prevalence	Clustering of cases in household–cases and household contacts are treated only
10–19% trachoma prevalence		In the absence of clustering—MDA for all children aged 6 months to 14 years in the community AND all household contacts treated.
		Annual screening
5-< 10% trachoma prevalence	At baseline, F & E only for 3 years then repeat SURVEY	Cases and household contacts 6 months– 14 years
		Annual screening
<5% trachoma prevalence	Two SURVEYS at 3 yearly intervals	Annual screen until <5% for 5 consecutive years (treat cases & household contacts found during the screen)

## Methods

Since 2006 when the National Trachoma Management Program was initiated, screening for trachoma and management has been consistently undertaken in three trachoma-endemic Australian jurisdictions, the NT, SA and WA. At program initiation, each jurisdiction identified Aboriginal communities considered to be at high risk of endemic trachoma from historical prevalence data and local knowledge, and over time, additional communities have been added to those considered at risk. In each designated community, regular screening rounds were undertaken over short time periods (generally several days), involving external teams working with local health staff. In most communities, 5–9 year olds were the focus of screening, as they were mostly in school, although children aged under 5 or 10–14 years were also screened if present at the time. Between 2010 and 2013 the estimated proportion of children resident in communities aged 5–9 that were screened for trachoma ranged from 57–71%.[6,8,11] Of those screened the WHO trachoma grading criteria[12] were used to diagnose and classify trachoma. Data from each community screened were collected on standardised data collection forms and included the numbers of children screened (in age groups 1–4, 5–9, 10–14 years), with active trachoma and with clean faces. The treatment strategy undertaken in the community and, from 2011, the numbers of household members and other community members treated, were also recorded. Data from screened communities have been presented in annual reports by the National Trachoma Surveillance and Reporting Unit and form the basis for analyses reported here.[6]

### Analyses

De-identified community-based data were obtained for each year from 2007, when comprehensive data collection began, through to 2013. As the majority of trachoma screening in communities was undertaken through primary school programs targeting 5–9 year olds, we restricted analyses of prevalence to this age group. The unit for analysis was a single episode of screening in 5–9 year olds within a single community. Community trachoma prevalence was estimated by dividing the number of 5–9 year olds with active trachoma during a screening round by the number screened. The treatment strategy adopted for a community in a given year was classified into one of four categories according to what was reported in the national database: no treatment; “active”cases only treated; “household” treatment under which active cases and their households members were treated; and “community-wide” treatment which covered both whole-of-community treatment (also known as “mass drug administration”) and a strategy under which active cases, household members and all children aged <15 years in the community were treated. For all strategies, the treatment administered for those over 6 months of age was a single weight-based dose (20mg/kg) of oral azithromycin [9].

Descriptive analyses by calendar year, examining all communities with eligible screening episodes were conducted. From 2011 onwards treatment coverage in communities for which household or community-wide treatment strategies were recorded was calculated by summing the population aged 0–14 years recorded as treated with azithromycin, and dividing by the total estimated population aged 0–14 years according to both census[13] and local health worker community population estimates.

To compare treatment strategies, we undertook two analyses. First we identified all communities for which data on trachoma prevalence in 5–9 year olds were available for pairs of consecutive calendar years. For each such pair of years, we estimated the change between the years in community prevalence, by simple difference. We then grouped communities by the first year of the consecutive pair and by the treatment strategy recorded in that year, and calculated a combined estimate of change for each treatment strategy using a random effects meta-analysis.[14] Second, for each community with at least two years of screening data, regardless of whether they were consecutive, we compared the change in prevalence from the first and final year of available data according to broad categories of treatment strategy (never treated, any non-community-wide treatment, treated at least once with community-wide), using the same meta-analytic method. As the treatment strategy used was strongly influenced by trachoma prevalence,[9] we conducted sensitivity analyses restricting communities to those with moderate prevalence (≥5% to <20%) at the start of the interval. We also stratified results by the two jurisdictions contributing the majority of data (NT and WA), and by community size (<250 versus ≥250 people) based on 2011 Australian census estimates.[13] Finally we conducted post-hoc analyses only including data collected for years 2007 to 2010 with the goal of differentiating secular trends in trachoma prevalence from effects of treatment. We used RevMan 5.5 software to estimate absolute differences in trachoma prevalence and SAS (version 9.3) for estimates of trachoma prevalence (function was unavailable in RevMan). The DerSimonian and Laird random effects model was used to obtain pooled estimates of risk difference, using the Mantel-Haenszel method to estimate the variation between studies. We estimated the combined prevalence using an exact likelihood approach.[15]

Administrative approvals for the data collection and analyses reported here were provided by the health departments of the three jurisdictions involved. Ethical approval was by the University of New South Wales Human Research Ethics Committee (ref 9-14-042).

## Results

We identified 914 screening episodes from 215 unique remote Aboriginal communities with children aged 5–9 years screened at least once between 2007 and 2013. Of the 215 communities, the majority were in the NT (n = 90; 42%) and WA (n = 99; 46%). There were 33 communities screened only once, 46 had 2–3 episodes, 59 had 4–5 episodes, and 77 had 6–7. The communities screened less frequently were more likely to have been screened for the first time more recently, with the median year of screening for communities with 3 or fewer years of screening data being 2012 compared to 2010 for those with four or more years of data.

Table 2 shows the number of communities screened each year, the proportion of communities screened from each of the three jurisdictions, the median number of children screened, the trachoma prevalence in 5–9 year olds and treatment strategies used. Biannual treatment (a second dose of antibiotics administered in the same year) was recorded following 1% of screening episodes. As biannual treatment was not unique to a particular treatment strategy, and numbers were small, we did not include this as a separate treatment classification. In general, the number of communities screened increased until 2013 when the NT adopted the revised guideline for screening[10] which recommends that screening in communities with high trachoma prevalence takes place every 3 years rather than annually. The median number of 5–9 year old children screened per community remained relatively stable over the seven years numbering about 20 (IQR 10–38). From 2008 to 2013, the proportion of communities with no trachoma detected increased (from 22.8% to 60.3%) while the proportion of communities with trachoma prevalence above 5% decreased (from 67.5% to 27.6%). In communities with trachoma detected, the median prevalence also decreased, from 23.1% to 8.9%. There was an increase in the proportion of communities not treated from 25.7% in 2008 to 56.0% in 2013, while from 2009 there was a fall in the number of communities treating ‘active’ cases only.

**Table 2:** Description of communities screening 5–9 year olds for trachoma between 2007 and 2013, Australia.

							Proportion using each treatment strategy†(%)			
Year	Number of communitiesscreened	Proportion from NT/SA/WA (%)	Median number of children 5–9 years screened in a community (IQR)	Proportion of communities with no trachoma detected in 5–9 years (%)	Proportion of communities with endemic (≥5%) trachoma in 5–9 years (%)	Median trachoma prevalence among 5–9 years in (%) communities with trachoma detected (IQR)	None	Active	House-hold	Community-wide
2007	121	49.6/6.6/43.8	17 (6–32)	39.7	54.6	19.3 (9.8–34.8)	62.8	10.7	37.2	9.1
2008	114	36.2/9.7/54.1	21 (11–38)	22.8	67.5	23.1 (9.1–43.5)	25.7	15.9	40.7	17.7
2009	126	42.1/10.3/47.6	20 (11–39)	31.8	58.7	18.8 (7.1–33.3)	36.8	20.8	31.2	11.2
2010	131	48.9/0.8/50.4	22 (11–43)	34.4	53.4	16.7 (5.9–33.3)	38.2	16.8	35.9	9.2
2011	156	43.6/12.2/44.2	22 (9–37)	47.4	40.4	10.0 (5.7–20.0)	41.3	1.9	46.5	10.3
2012	150	47.1/10.7/42.7	22 (11–37)	57.3	28.3	8.9 (3.8–14.3)	45.4	2.1	39.0	13.5
2013	116	27.6/12.9/59.5	17 (9–35)	60.3	27.6	8.9 (4.3–14.3)	56.0	0	33.6	10.3
**Total**	**914**	**41.9/12.1/46.1**	**20 (10–38)**	**42.6**	**46.3**	**14.1 (5.9–29.5)**	**43.7**	**9.4**	**35.4**	**11.5**

^†^ See methods for definition of treatment strategies.

For 2011 and 2012 (Table 3), using local estimates of the population size, treatment coverage among 120 communities that reported having used a “household” treatment strategy was 11.9% (95%CI 11.4–12.5%) while for the 33 communities using a “community-wide” treatment strategy, treatment coverage was 75.0% (95%CI 73.5–76.4%). The estimates were similar when Census population estimates[13] were used.

**Table 3:** Treatment coverage among 0–14 year olds according to reported treatment strategies, 2011 and 2012.

Treatment strategy	Source of denominator	Communities* (N)	Population treated/Estimated total population (n/N)	Coverage (%)
Household	Census	80	1231/10651	11.6
Household	Community estimate	120	1470/12309	11.9
Community-wide	Census	32	3055/3894	78.4
Community-wide	Community estimate	33	2574/3431	75.0

*Community counts may include some communities twice as episodes combine records from 2011 and 2012.

After excluding the 33 communities with only a single year of screening data available, there remained 881 records from 182 unique communities; 77 (42.3%) were from the NT, 21 (11.5%) from SA and 84 (46.2%) from WA. Of 121 communities that applied a treatment strategy in more than one of the years observed, 89 were recorded as having changed strategies over the time period, 30 communities used only household treatments, two used only community-wide treatments, and none applied the “active” case only strategy more than once.

**Fig 1:**
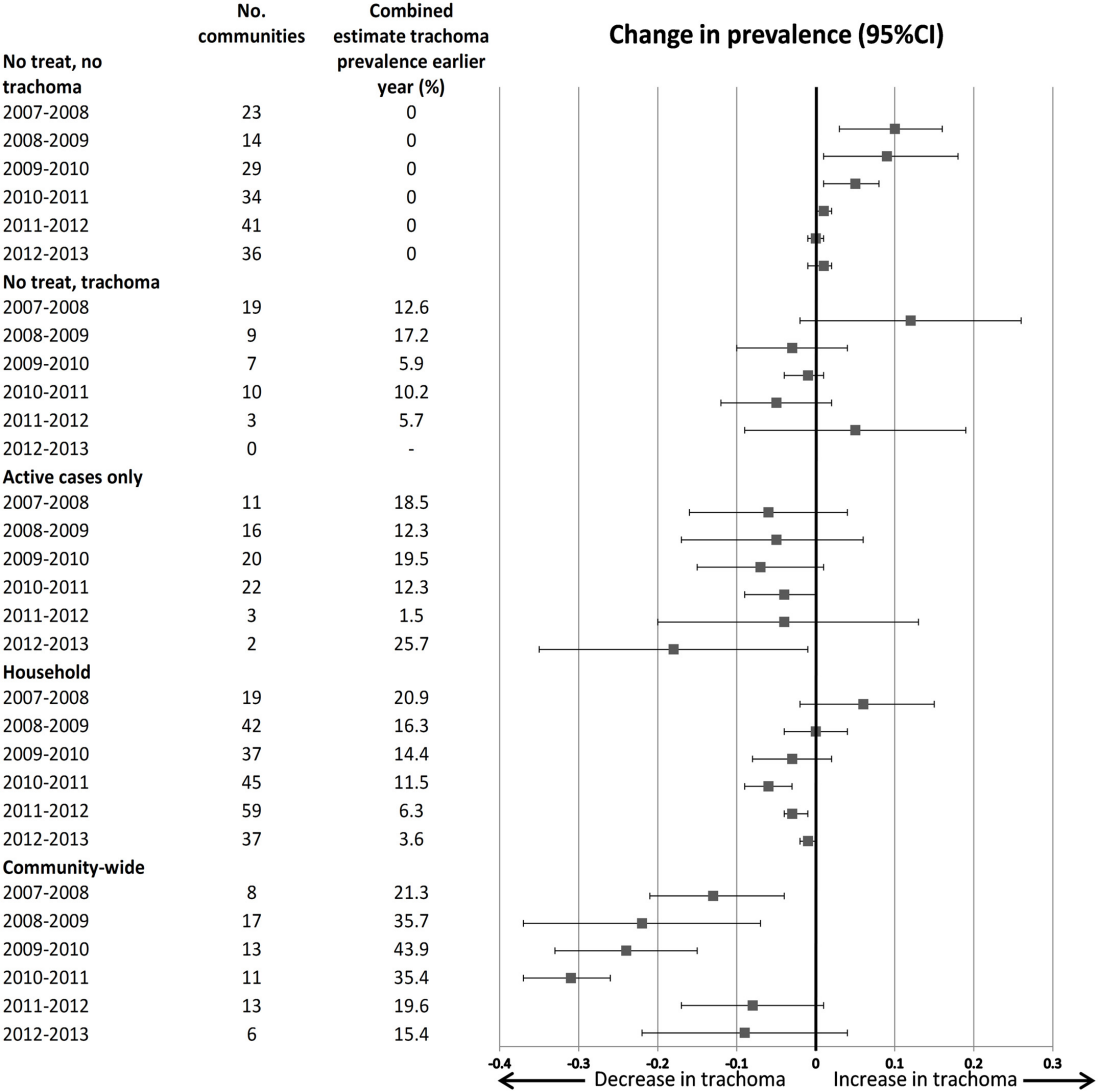
Combined estimate of change in community trachoma prevalence between years according to treatment strategy, all communities.

Fig 1 shows the estimated change in trachoma prevalence between pairs of successive years, according to the treatment strategy used in the initial year of the pair. Communities recorded as receiving no treatment are separated according to whether they had trachoma detected or not in the initial year of the pair. In the earlier years of the program (2007–2010) for communities without trachoma detected and not treated, there was a significant increase in prevalence between pairs of years (e.g. absolute risk increase of 10% [95%CI 3% to 16%] from 2007 to 2008) but after 2010 there was no substantial change. The number of communities that were not recorded as having been treated despite trachoma being detected decreased over time. In these communities, trachoma prevalence between years did not change significantly between pairs. For all categories of treated communities (active case only, household, or community-wide) there was a reduction in estimated trachoma prevalence between the pairs of years; in most years this was not statistically significant. The largest absolute reductions in trachoma prevalence were in communities that were recorded as having received community-wide treatment, with point estimates ranging from -8% to -31%; the reductions were only statistically significant for the years 2007–2011. These communities receiving community-wide treatment also had the highest prevalence in the earlier of the paired years (range for combined estimates 15.4%-43.9%).

The majority of communities (n = 176) had annual records of trachoma screening that included at least two years of the eligible period (2007 to 2013), including 68 with data for 7 consecutive years, 47 with 6 years, 25 with 5 years, 12 with 4 and 24 with less than 4. Based on the treatment strategies used between the first and final year of data recorded, communities were grouped into three categories (Table 4): those never treated; those treated but never with community-wide strategies (i.e. only active case or household treatment); and those treated at least once using a community-wide strategy.

**Table 4:** Distribution of treatment strategies used across all years of available data, in communities by whether communities ever had community-wide treatment.

	No. communities	Treatment strategies used† (%)			
		None	Active	Household	Community-wide
**Never treated**	23	100	0	0	0
**Treated but never with community-wide strategy**	88	41.6	13.1	45.3	0
**Treated at least once with community-wide strategy**	65	29.1	11.0	32.2	27.6

^†^ See methods for definition of treatment strategies.

**Fig 2:**
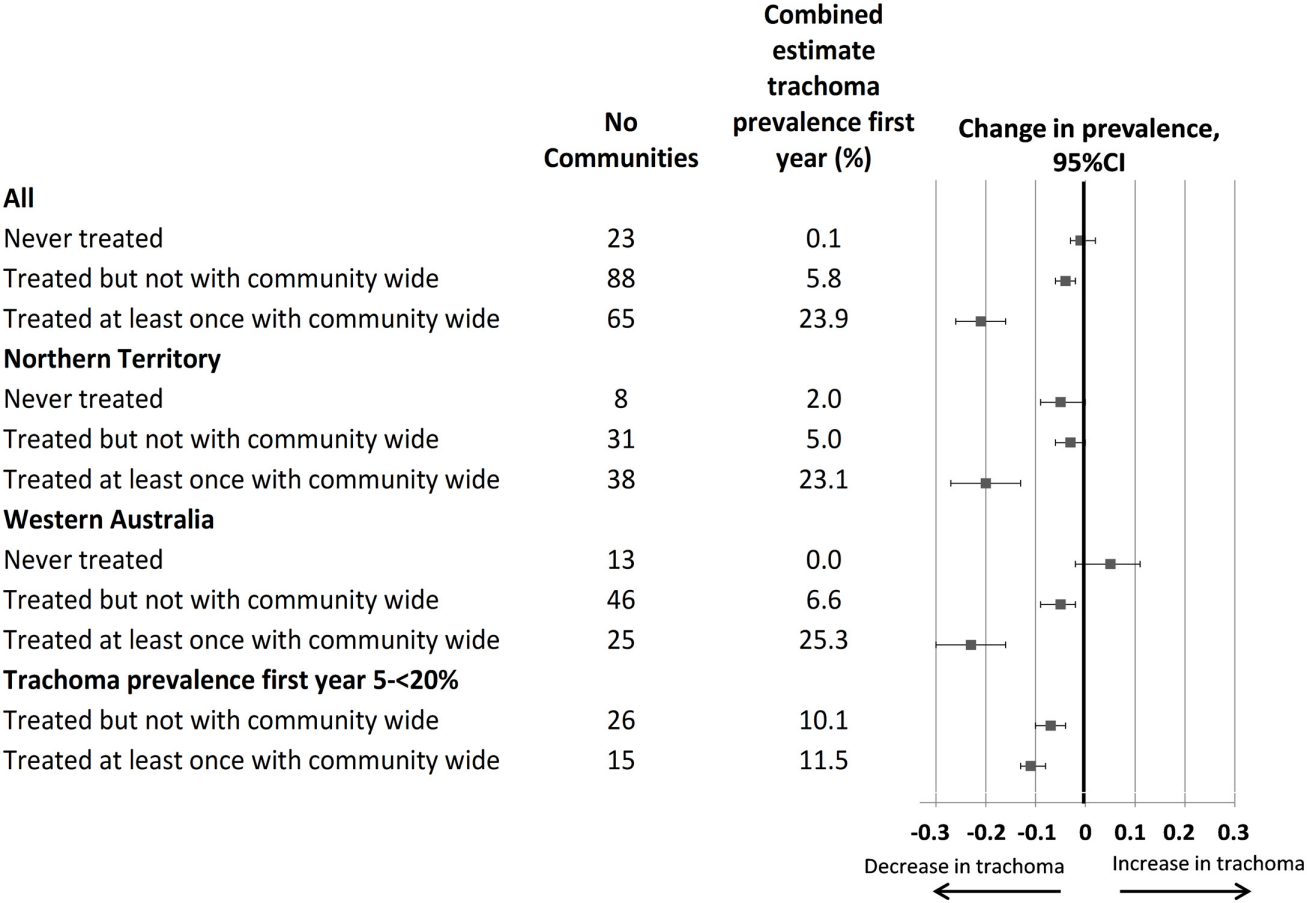
Combined estimate of change in community trachoma prevalence from first to last year of data collection, according to treatment strategy, by jurisdiction, and in subgroup of initial prevalence.

Fig 2 shows the estimated change in trachoma prevalence between the first and final years of data, by the three classifications of communities in Table 4. For communities never recorded as receiving azithromycin for trachoma, the estimate of prevalence in the first year of screening was 0.1% and the estimated absolute reduction over time 0% (-3% to 2%). For communities that received only active case and household strategies, the prevalence in the first year of screening was 5.8% and the reduction over time -4% (-2% to -6%). Among communities treated at least once with community-wide strategies, initial prevalence was 23.9%, and the reduction -21% (-16% to -26%). These patterns were similar when communities from the NT or WA were considered separately.

When we restricted analyses to communities with moderate trachoma prevalence (5-<20%) in their first year of screening (Fig 2) we found that there was only a small difference between those that had received community-wide treatment and those that had not, with reductions of -11% (-8% to -13%) and -7% (-5% to -10%) respectively, from a similar initial prevalence (11.5% and 10.1% respectively). Restricting analyses to communities with at least four years of screening data did not change the findings, and we found no differences in treatment effects when we compared smaller (N<250 people) to larger sized communities (N≥250 people).

Analyses of data restricted to 2007–2010, the period during which there were substantial increases in trachoma prevalence in previously trachoma-free communities that were untreated (see Fig 1), are shown in Table 5. In the communities that were never treated, and in those treated but not with a community wide strategy, there was no significant fall in trachoma prevalence, while communities with at least one community-wide treatment had a 14% (95%CI 9 to 20%) decline in trachoma prevalence. When we further restricted analyses to communities with moderate trachoma prevalence (5-<20%) we found that those with at least one community-wide treatment had a significant reduction in prevalence but those treated with more targeted strategies did not.

**Table 5:** Combined estimate of change in community trachoma prevalence from first year of data collection until 2010, according to treatment strategy.

	No. of communities	Change in prevalence (95%CI)
**All**		
Never treated	26	0.01 (-0.03 to 0.04)
Treated but not with community wide	73	0.00 (-0.03 to 0.03)
Treated at least once with community wide	40	-0.14 (-0.20 to -0.09)
**Trachoma prevalence first year 5-<20%**		
Treated but not with community wide	21	0.02 (-0.05 to 0.09)
Treated at least once with community wide	9	-0.07 (-0.03 to -0.11)

## Discussion

In this investigation of the relationship between different community treatment strategies for trachoma control and long-term changes in trachoma prevalence, we found that in high prevalence communities, community-wide administration of azithromycin, or MDA, was associated with a substantially reduced trachoma prevalence after one year or more. In settings with moderate trachoma prevalence (5-<20%), more limited strategies were equally effective in the longer term. As discussed in more detail below, this finding may have particular relevance for countries moving towards elimination, but with localised areas of moderate prevalence remaining.

Observational studies have shown that a single MDA of azithromycin in communities with endemic trachoma results in substantial reductions at one year in trachoma prevalence in both hyperendemic (>20% prevalence)[16,17] and moderately endemic (5-<20% prevalence)[3,18] communities. Recent trials have compared annual versus biannual mass azithromycin administration in high prevalence communities, but the trials have not consistently found that larger or more sustained reductions can be achieved with more frequent treatment.[19,20] There are few reports comparing different treatment strategies in moderate prevalence settings. One study found that targeted (household) treatment may be as effective as mass treatment of all children but only had follow-up for 6 months.[21] Another suggested a single mass drug administration may be effective in sustaining a reduction in trachoma prevalence over many years[3] and another suggested that treatment that was not community-wide led to re-infections.[22] Our findings regarding ‘community-wide’ treatment (Fig 1) over one year concur with the observational studies of mass drug administration showing that this approach is effective in substantially reducing trachoma prevalence in high prevalence settings. Our main analyses also suggest that in more moderate prevalence settings, targeted treatment strategies (mostly ‘household’ strategies, whereby active cases and all members of their household were treated with azithromycin), were also associated with reduced trachoma over a year and for longer periods (Figs 1 and 2).

In the paired-year analyses (Fig 1), among communities that had no trachoma detected at the start of the observation period, and were consequently not treated, we found that there were annual increases in prevalence between 2007 and 2010. However from 2011 onwards, prevalence remained at zero, i.e. no change. Given the mobility of people between Aboriginal communities,[23] this observation may be evidence that antibiotic treatment programs in communities with trachoma detected can have a “herd” effect, in that transmission to trachoma-free communities is prevented. It is also possible that this resulted from other components that are delivered as part of the SAFE strategy, such as promotion of facial cleanliness and environmental improvements.

As the paired-year analysis indicated no overall increase in trachoma prevalence in trachoma-free communities from 2010 onwards, we undertook analyses involving multiple years with the goal of distinguishing effects of treatment from temporal changes in trachoma. In these sensitivity analyses, only communities receiving community-wide treatment were found to have a reduction in trachoma prevalence (see Table 5). It is therefore possible that the trachoma reduction in moderate prevalence settings observed in our primary analyses may in fact have been a result of overall declines in trachoma burden rather than a result of targeted treatment.

This was an observational study using routinely collected surveillance data.[5] While diagnosis was undertaken by specialised teams of health care workers following standard international guidelines, there may have been diagnostic error, to an extent that cannot be measured. Our analysis of impact was also limited by the absence of detailed data for all years on the level of treatment coverage achieved in each community. However for 2011 and 2012, data were available for the majority of communities and this indicated that coverage was substantially different between those communities reporting “household” compared to those reporting “community-wide” treatment. There may also be factors that differed between communities or changes over time that were not measured but were associated with treatment strategy and therefore could have affected the summary estimate of difference in trachoma prevalence observed. For example, we did not include in our analyses other factors that may contribute to changes in trachoma prevalence.[1] Facial cleanliness, and facial cleanliness promotion (‘F’ in the SAFE strategy) was reported in the communities screened, but not considered to be sufficiently standardised or validated to use in the analyses presented here.[24] Data on environmental factors (‘E’ in the SAFE strategy) such as improved housing conditions, or the availability of swimming pools, were limited and inconsistent.[25] Despite the absence of information on facial cleanliness and environmental factors, we do not have evidence to suggest that they linked to treatment status and thus had any potential to bias our results.

The strengths of our study are the use of annual trachoma screening data from all communities in the three jurisdictions with known endemic trachoma leading to a more comprehensive picture of not only the effects of different treatment strategies on single communities, but also programmatic effects on all communities in a real-world setting. We also had observations for the majority of communities over a significant period of time (at least four years) and were able to observe the effects of a targeted treatment strategy in a moderate prevalence setting.

In summary, our study supports current evidence that recommends mass azithromycin administration to reduce trachoma prevalence in high prevalence settings. We also found that less intensive treatment with a “household” strategy in moderate prevalence communities (5-<20%) may be associated with reductions in prevalence similar to mass drug administration. This finding may have implications for countries that are moving to lower levels of endemic trachoma and wish to reduce the amount of azithromycin being used. The strategy does however have the requirement that individual examination must take place, to determine which households have affected members. If a targeted approach is to be considered, trials and health economic analyses are required to determine which option may be more cost-effective in particular programmatic and community contexts.[26] Finally our results also suggest that trachoma program implementation can reduce trachoma prevalence in communities not specifically targeted (“herd effects”) and thereby contribute to reducing trachoma transmission.

## Supporting Information

S1 Checklist STROBE Checklist. (DOCX)
